# The role of phosphatidylserine on the membrane in immunity and blood coagulation

**DOI:** 10.1186/s40364-021-00346-0

**Published:** 2022-01-15

**Authors:** Jiao Wang, Changxin Yu, Junyi Zhuang, Wenxin Qi, Jiawen Jiang, Xuanting Liu, Wanwei Zhao, Yiyang Cao, Hao Wu, Jingxuan Qi, Robert Chunhua Zhao

**Affiliations:** 1 School of Life Sciences, Shanghai University, 99 Shangda Road, Shanghai, 200444 China; 2 Institute of Basic Medical Sciences, Chinese Academy of Medical Sciences, School of Basic Medicine, Peking Union Medical College, No. 5 Dongdansantiao, Beijing, 100005 China; 3 Centre of Excellence in Tissue Engineering, Chinese Academy of Medical Sciences, Beijing, China; 4 Beijing Key Laboratory of New Drug Development and Clinical Trial of Stem Cell Therapy (BZ0381), Beijing, China

**Keywords:** Phosphatidylserine, GLA domain, Discoidin-like C2 domain, IgV-like domain, TAM, TIM, Blood coagulation (hemostasis), Immunity, Apoptosis, COVID-19

## Abstract

The negatively charged aminophospholipid, phosphatidylserine (PtdSer), is located in the inner leaflet of the plasma membrane in normal cells, and may be exposed to the outer leaflet under some immune and blood coagulation processes. Meanwhile, Ptdser exposed to apoptotic cells can be recognized and eliminated by various immune cells, whereas on the surface of activated platelets Ptdser interacts with coagulation factors prompting enhanced production of thrombin which significantly facilitates blood coagulation. In the case where PtdSer fails in exposure or mistakenly occurs, there are occurrences of certain immunological and haematological diseases, such as the Scott syndrome and Systemic lupus erythematosus. Besides, viruses (e.g., Human Immunodeficiency Virus (HIV), Ebola virus (EBOV)) can invade host cells through binding the exposed PtdSer. Most recently, the Corona Virus Disease 2019 (COVID-19) has been similarly linked to PtdSer or its receptors. Therefore, it is essential to comprehensively understand PtdSer and its functional characteristics. Therefore, this review summarizes Ptdser, its eversion mechanism; interaction mechanism, particularly with its immune receptors and coagulation factors; recognition sites; and its function in immune and blood processes. This review illustrates the potential aspects for the underlying pathogenic mechanism of PtdSer-related diseases, and the discovery of new therapeutic strategies as well.

## Background

**Fig. 1:**
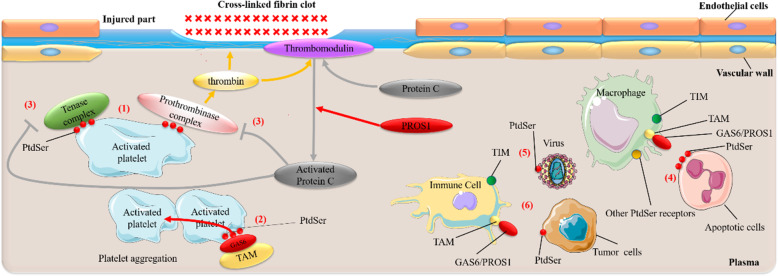
The role of phosphatidylserine in immunity and blood coagulation. In the blood coagulation system, (1) PtdSer exposed on activated platelets can bind to the Tenase complex and Prothrombinase complex to promote the generation of thrombin. To form a clot at the injured site to achieve a hemostatic effect, thrombin activates downstream signaling pathways [28–32]. (2) PtdSer exposed on activated platelets can bridge with TAM receptors through the GAS6 ligand to promote platelet aggregation to maintain thrombosis and platelet stability [33]. (3) The thrombin generated through (1) can also bind to thrombomodulin to catalyze the production of Activated Protein C from Protein C. Subsequently, they inactivate Tenase complex and Prothrombinase complex, inhibiting blood coagulation. During this process, PROS1 support the anticoagulant activity of Activated Protein C [28–32]; In the immune system, (4) PtdSer exposed on the surface of apoptotic cells can be recognized and bound by PtdSer receptors, such as TIM, TAM, on the surface of phagocytes, leading to phagocytosis of apoptotic cells [19, 34, 35]. (5) The PtdSer exposed on the surface of some viruses, such as the Ebola Virus, Dengue Virus, can binds to PtdSer receptors on immune cells (i.e., TIM, TAM, etc.), allowing the virus to invade hosts [9–15]. (6) PtdSer exposed on tumor or cancer cells can also bind to PtdSer receptors (e.g., TIM, TAM) expressed on immune cells to trigger immunosuppressive pathways and ultimately promote immune escape [19–22]

In eukaryotic cells, the distribution of lipids on biological membranes is asymmetric [1]. Glycosphingolipid, sphingomyelin, and phosphatidylcholine (PtdCho) mainly distribute on the outer leaflet, while aminophospholipids such as phosphatidylethanolamine (PtdEtn) and phosphatidylserine (PtdSer) mostly distribute on the inner leaflet. PtdSer, as an aminophospholipid, its asymmetry distribution on membranes is essential for various biological processes [2]. As Immune and blood coagulation play a vital role in the human body and within them, PtdSer is essential for the normal execution of specific immune and coagulation processes. Once the functions of PtdSer are abnormal, they will cause many diseases, including Scott syndrome [3], Systemic lupus erythematosus (SLE) [4], Essential hypertension (EH) [5], Hemophilia A [6], Alzheimer’s disease (AD) [7], Human Immunodeficiency Virus (HIV) [8], Ebola virus (EBOV) [9–12], Dengue virus (DENV) [13–15] and Respiratory Syncytial Virus (RSV) [16–18], and certain tumors or cancers [19–22]. It was also speculated that PtdSer might be a potential mechanism or participant of inflammation and coagulation abnormalities in COVID-19 [23, 24]. Therefore, studies in Ptdser, its interacted molecules and their structure features, are of significance, not only an aspect to further understand these diseases but also a potential therapeutic target. Additionally, miles of researches have focused on blood coagulation and inflammation to elucidate the close relationship between them. Molecules that affect blood coagulation usually affect inflammation, and vice versa [25–27]. The Receptor tyrosine kinases TAM family, protein S (PROS1), GLA domain, and C2 domain, binding to PtdSer, all participate in both blood coagulation and immune processes, which means a crucial role of Ptdser in the immune and blood coagulation system (Fig. 1). Therefore, this review focuses on the mechanism of PtdSer exposure during immunity and blood coagulation and how PtdSer interacts with immune receptors and coagulation factors. Meanwhile, this review as well as summarized some PtdSer-related diseases of coagulation or immunity, including COVID-19, in recent years.

## The mechanism of PtdSer exposure on apoptotic cells in the immune system

Apoptosis [36] is also called programmed death, which is the process of cell death caused by the internal and external factors triggering the death program in the cell. There are two main signaling pathways at the beginning, one is the endogenous apoptosis pathway, which is regulated by mitochondria [37]; the other is the exogenous apoptosis pathway, which is regulated by death receptors [38]. Once apoptosis is activated, cross-talking occurs in these two signal pathways [39], and eventually, they will activate the caspase family proteins (cysteine-containing aspartate-specific proteases). These caspase family members are the executors of apoptosis, resulting in PtdSer exposure in the cell membrane during the pre-apoptotic stage [40–44], which ultimately lead to DNA fracture and protein degradation [45, 46]. Phagocytes, recognizing PtdSer on the surface of apoptotic cells by its receptors [19, 35, 47, 48], subsequently, engulf the apoptotic cells to avoid inflammation [49].

In normal cells, PtdSer is maintained in the plasma membrane’s inner leaflets by flippases such as ATP11A and ATP11C [42]. However, during immune and coagulation processes, ATP11A and ATP11C can be inactivated by other factors like Ca^2+^ or caspase [41, 42]. Simultaneously, scramblase, such as Xk-related Protein 8(Xkr8) and Transmembrane protein 16F (TMEM16F), will be activated by the same factors, causing PtdSer exposure to the plasma membrane’s outer leaflets [40, 50].

P4-ATPases, belong to flippase, at least fourteen are encoded in mammals [51–54] and play a crucial role in establishing and maintaining phospholipid asymmetry across the membranes. They transport phospholipids across the membranes rely on ATP hydrolysis particularly when PtdSer and PtdEtn are substrates of P4-ATPase [55]. CDC50 family proteins are essential for P4-ATPase to transfer PtdSer from the plasma membrane’s outer leaflet to the inner leaflet [42]. ATP11C and ATP11A are identified as flippase on the plasma membrane [41, 42]. Ca^2+^ is required to inhibit the activity of flippase (ATP11C and ATP11A) for PtdSer exposure on activated platelets and the cleaved caspase is so for PtdSer exposure on apoptotic cells, and thereby inhibiting PtdSer inversion [41, 42].

Scramblase controls the disruption of lipid asymmetry, catalyzing the non-ATP-dependent bidirectional movement of lipids on the plasma membrane of eukaryotic cells [56]. It includes two protein groups, one is the caspase-dependent Xk family protein [40], and the other is the Ca^2+^-dependent transmembrane protein 16 (TMEM16) family protein [50]. In eukaryotic cells, two proteins, Xkr8 and TMEM16F, are involved in PtdSer exposure, for which Xkr8 is responsible on the surface of apoptotic cells, while TMEM16F is on the surface of activated platelets [40, 50].

### Caspase activates the activity of Xkr8 and inactivates ATP11C and ATP11A

**Fig. 2:**
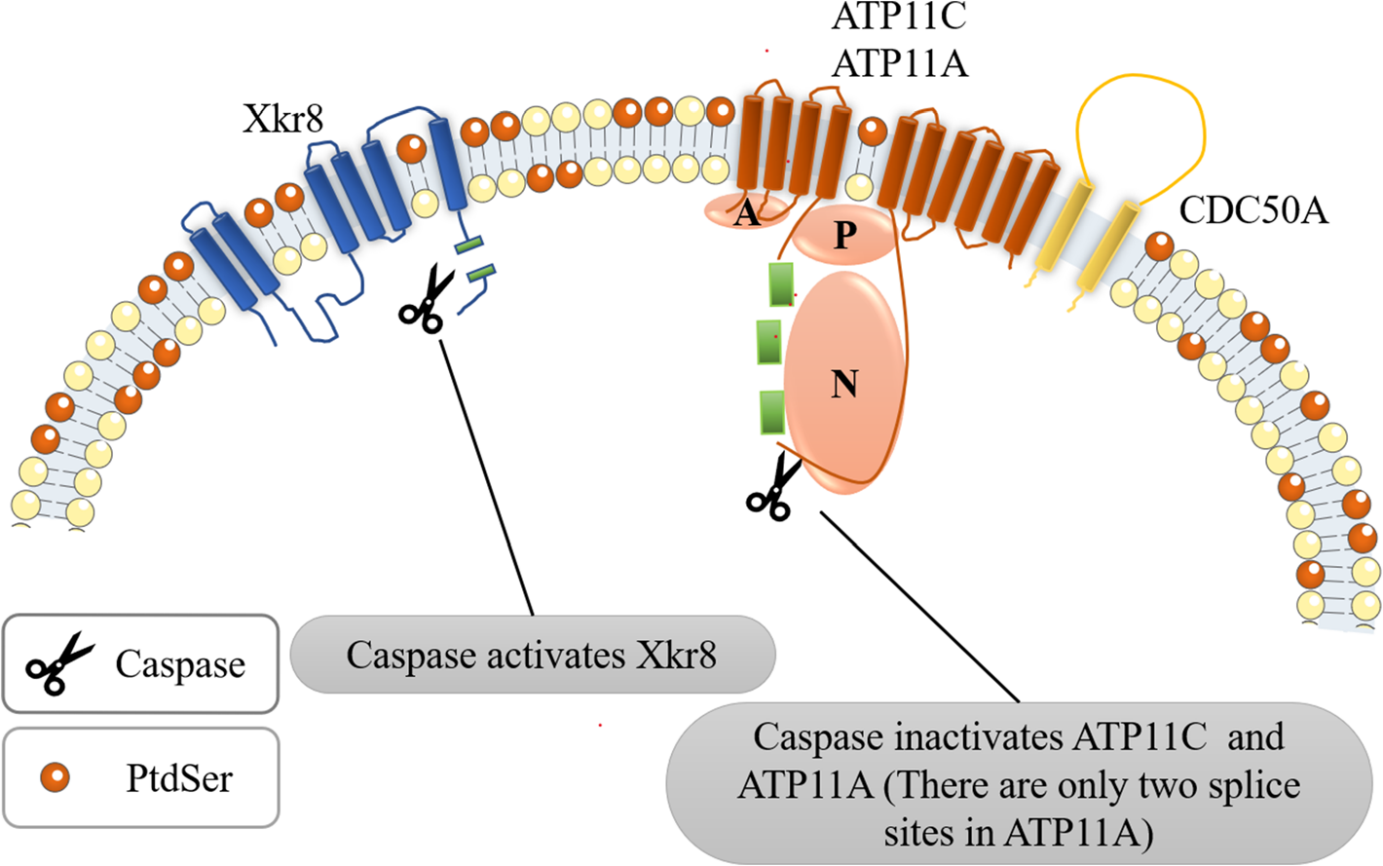
The process of phosphatidylserine exposure on the surface of apoptotic cells. This figure schematically shows the structure of caspase-dependent scramblase Xkr8 and flippase (ATP11A /ATP11C related to CDC50A). When cell apoptosis occurs, caspase 3 or caspase 7 cleaves Xkr8 to activate its scramblase activity [20, 21]. Meanwhile, caspase 3 or caspase 7 cleaves ATP11A and ATP11C to inactivate their flippase activity, resulting in the loss of lipid asymmetry and PtdSer exposure. Then the macrophages will engulf the apoptotic cells. This form of PtdSer exposure is irreversible [22]

A large amount of PtdSer is exposed to the apoptotic cell membrane [57]. Once entering the apoptotic or necrosis state [58], apoptotic cells undergo specific surface changes to induce professional or non-professional phagocytes to bind to and then engulf them [58]. The most remarkable surface change is the surface exposure of PtdSer, caused by the loss of plasma membrane asymmetry, in which scramblase Xkr8, caspase 3/7, ATP11A, ATP11C, and CDC50A play a synergistic role (Fig. 2), [40–44].

Xkr8, containing 6 transmembrane regions, is ubiquitously expressed in various tissues and, as a scramblase, is essential for the eversion of PtdSer during apoptosis [40, 44]. Xkr8 has a conservative caspase recognition site in its C-terminal tail region that can be cleaved by caspase 3/7, which is essential for activating its scramblase activity [40]. After Xkr8 is cleaved and activated by caspase, it aggregates with basigin (BSG) or neuroplastin (NPTN) in Ig superfamily. This kind of polymerization is formed by two Xkr8 molecules and two BSG or NPTN molecules to form a heterotetramer, which together complete the function of scramblase for phospholipid scrambling [43].

Caspase can inactivate ATP11A and ATP11C as well. According to cryo-electron microscopy (Cryo-EM), they contain ten transmembrane regions [59]. These phospholipid flippases contain caspase recognition sites, three cleavage sites in the “N” domain (Nucleotide-binding domain) of ATP11C [42], and two cleavage sites in the “P” (phosphorylation domain) and “N” domains of ATP11A [41]. Once these sites are mutated, ATP11C and ATP11A cannot activate their flippase activity. In the apoptosis process, these sites of ATP11C and ATP11A are cleaved by caspase 3/7, to inactivate ATP11C and ATP11A [42], finally leading to PtdSer exposure. This caspase-mediated flippase inactivation is necessary for PtdSer exposure on apoptotic cells [41, 42].

### Diseases caused by reduced PtdSer exposure on the surface of apoptotic cells

Lupus-like autoimmune disease (or systemic lupus erythematosus (SLE)) is a typical example [4]. The patients’ immune system produces autoantibodies to attack their own cells and tissues, causing inflammation and organ damage [60]. The study of Mahiru Kawano [4] found that Xkr8 deficiency severely delayed PtdSer exposure on the surface of apoptotic splenocytes, neutrophils, and thymocytes. The exocytosis of these cells is suppressed. This study shows that Xkr8-mediated PtdSer exposure in apoptotic lymphocytes and senescent neutrophils is a key step in apoptosis. After PtdSer exposure, these cells are engulfed, preventing the release of toxic substances from dying or senescent cells. Once Xkr8 deficiency results in reduced PtdSer exposure, it will activate the immune system, leading to SLE [4].

## Immune receptors for PtdSer

Macrophages only engulf apoptotic cells, but not healthy cells [61]. This specificity depends on the “eat-me” signal on the surface of apoptotic cells [62]. The most representative signal is Phosphatidylserine (PtdSer) signal, the main “eat-me” signal [19]. Due to PtdSer exposure, apoptotic cells can be quickly and effectively recognized and eliminated by phagocytes. On the contrary, once the clearance fails, the apoptotic cells may enter the secondary necrotic stage, cause the phagocytes, and release pro-inflammatory cytokines and trigger inflammation [49].

There are multiple PtdSer receptors on immune cells. The mechanism for recognition of PtdSer by its receptor is crucial for the the process of endocytosis of immune cells. PtdSer receptor families are multiple, of which two types have been identified as PtdSer-sensing receptors TAM receptor protein tyrosine kinases family (TYRO3, AXL, and MER) and TIM family (T cell/transmembrane, immunoglobulin, and mucin) [35, 47, 48]. Additionally other receptors on immune cells include αvβ3 or αvβ5 integrins [63, 64], CD300a [14, 65], BAI1 [66], Stabilin [67, 68], RAGE [69], LOX-1 [70], etc. Most of them all can recognize PtdSer exposure on the surface of apoptotic cells, and thus endocytosis, while some of the downstream mechanisms are still unknown.

### TAM receptors family recognizes PtdSer in the immune and blood coagulation systems

TAM receptors include TYRO3, AXL, and MER, which are receptor protein tyrosine kinases (RPTKs or RTKs) on the cell surface [71]. RTKs are transmembrane glycoproteins, serve as both receptors and enzymes, binding to ligands and phosphorylate tyrosine residues of target proteins. RTKs binding to homologous ligands form dimers or tetramers, thereby catalyzing the receptor autophosphorylation and tyrosine phosphorylation of the substrates of RTKs. It transmits extracellular signals to the cytoplasm and activates many signal transduction pathways in the cell [72, 73].

TAM-RTKs (hereafter referred to as TAM) are members of the RTKs family. They are expressed by many cells, such as Macrophages [74], Dendritic Cells [74, 75], Antigen presenting cell [74], immature natural killer (NK) cells [76], Cerebellar Purkinje cells [77], Hippocampal dentate gyrus [77], Retinal pigment epithelium (RPE) cells [78], and Sertoli cells [79] and play a vital role in hemostasis and anti-inflammatory [25, 80, 81].

#### TAM, GAS6/PROS1 and PtdSer in immune system

**Fig. 3:**
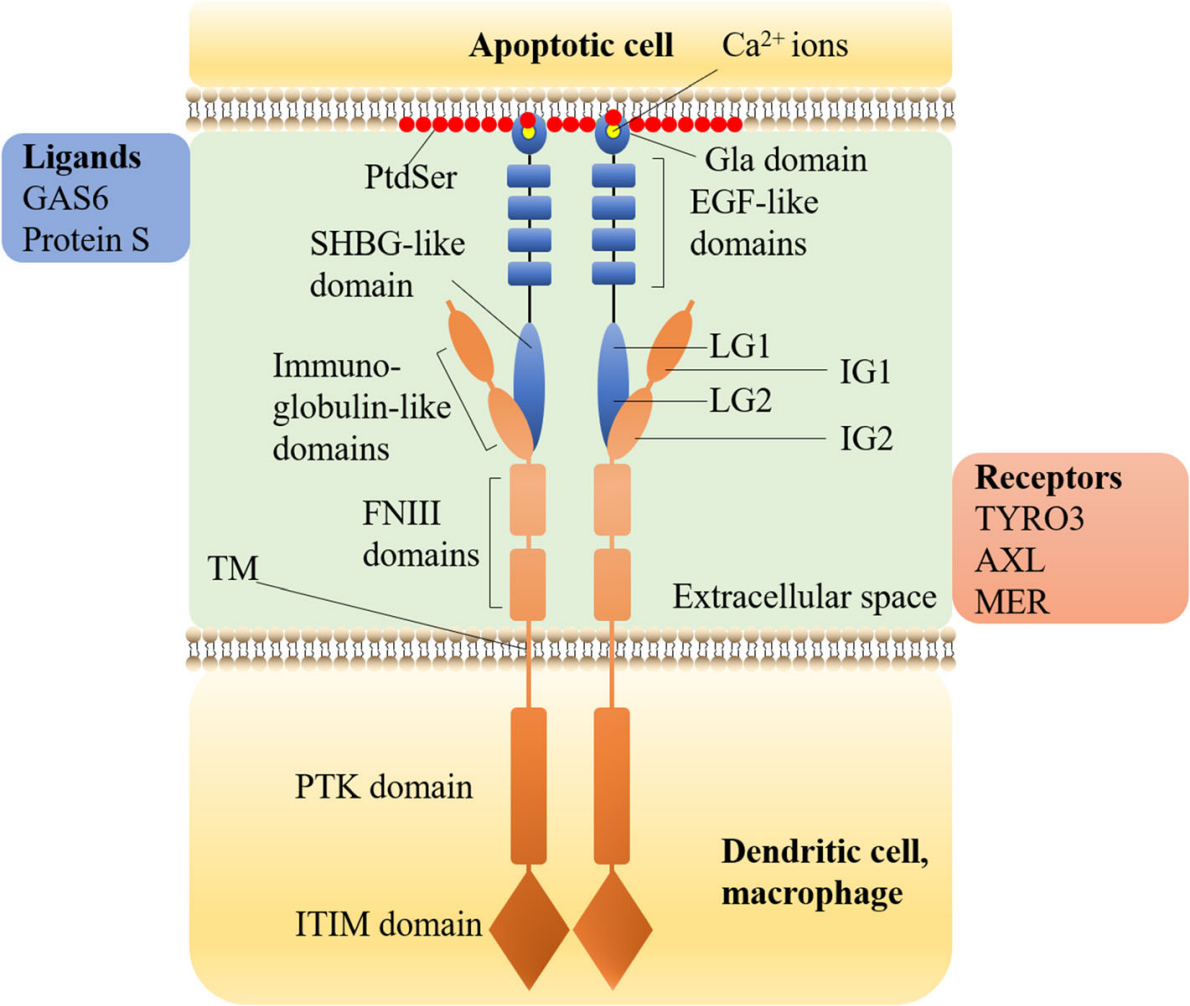
The Structure of TAM, GAS6/Protein S and how they bind to PtdSer. TAM is a single transmembrane receptor (the orange part in the figure). From N-terminal to C-terminal (from top to bottom in the figure), there are three parts:1, the extracellular domain, including two IG domains (IG1, IG2) and two FNIII domains; 2, one single TM; 3, the intracellular domain, including one conserved PTK domain, one autophosphorylation site, and one ITIM domain [23–27]. The intracellular PTK domain of TAM is the functional domain. TYRO3, AXL, and MER of TAM family all have phosphorylation and autophosphorylation sites, which can activate the corresponding signaling pathways by phosphorylation of downstream target proteins. TAM does not directly bind to the PtdSer exposed on the cell surface but relies on GAS6 or PROS1 as a ligand [28]. GAS6/PROS1 consists of three parts (the blue part in the figure). From N-terminal to C-terminal (from top to bottom in the figure), there are N-terminal GLA domain, four EH -like domains, and C-terminal SHBG domain [7, 29, 30]. The TAM receptor binds to the C-terminal SHBG domain of GAS6/PROS1 through two N-terminal IG1 and IG2 in the extracellular domain. SHBG domain includes two tandem LG domains (LG1, LG2). The binding eventually leads to the activation of TAM receptor tyrosine kinase. GAS6/PROS1 binds to the PtdSer exposed outside the cell through the N-terminal GLA domain. With the assistance of vitamin K, the structure of the GLA domain can be stabilized through the binding of about 7 essential Ca^2+^ ions (yellow ball at the top in the figure) [7, 31–34]. The specific mechanism of interaction between the GLA domain and PtdSer will be introduced in section 4.2

The immune system uses many methods to eliminate inflammation, one of which is to activate TAM for anti-inflammation. Only when TAM correctly recognizes and binds to PtdSer (Fig. 3) can it avoid inflammation caused by the failure to clear apoptotic cells, then play the anti-inflammatory effect of TAM [34, 49]. TAM does not directly bind to the PtdSer exposed on the cell surface but relies on growth-arrest-specific 6 (GAS6) or protein S (PROS1) as a ligand [82]. Both GAS6 and PROS1 are vitamin K-dependent proteins with similar structures (Fig. 3). Their C-terminal is used for TAM binding and phosphorylation to be used as TAM ligands [83]. Studies have found that the binding of GAS6/PROS1 to TAM does not depend on PtdSer and Ca^2+^. The optimal activation of TAM receptors for any ligand requires the presence of PtdSer and Ca^2+^ that bind to the GLA domain of the ligand. In the signal transduction process, PtdSer, γ-carboxylated GAS6/PROS1 ligand, and TAM receptor together constitute a complete TAM signal transduction module [84].

It is worth noting that GAS6 binds and activates all three receptors, while PROS1 binds and activates TYRO3 and MER but does not activate AXL [84]. Through receptor-ligand activation curves, the difference in TAM signal is from the two N-terminal Ig-like domains of the TAM receptor but not the difference in TAM kinase activity [84]. Most or all of GAS6 in cells and tissues in the body have specifically bound to AXL, showing AXL dependence. The affinity of GAS6: AXL ≥ TYRO3 >> MER [84].

After binding to PtdSer, TAM activates downstream signaling pathways:

(i) TAM receptors expressed on macrophages or dendritic cells can recognize PtdSer on the surface of apoptotic cells and activate phagocytosis by activating Rac1 (a GTPase) [85]. Rac1 is a member of Ras Homologue (Rho) GTPase. Rho GTPase mainly affects the remodeling of the cytoskeleton. Rac1 acts as a positive regulator of phagocytosis by inducing cytoskeleton rearrangement [80, 85–87].
(ii) TAM receptor on immune cells and GAS6/PROS1 ligand binding to PtdSer on apoptotic cells have direct anti-inflammatory activity that suppresses inflammatory cytokines and nuclear factor-κB (NF-κB) [19, 88]. For example, TAM prevents inflammation in our body by inhibiting the Toll-like receptor (TLR) and TLR-induced cytokine receptor cascade [75].. It has been shown that TLR signaling significantly reduces the expression of GAS6 in mouse macrophages by activating NF-κB, thereby further promoting TLR-mediated inflammation in a self-regulating manner [89]. Researches have shown that the MER receptor expressed on macrophages and dendritic cells has an inhibitory effect on the activation of NF-κB. This effect is MER-specific [90]. Also, GAS6-induced AXL activation inhibits TLR and type I interferon (IFN) receptor signal transduction by up-regulating the expression of SOCS1 and SOCS3 (Suppressor of cytokine signaling (SOCS) protein can inhibit the activity of TLR), thereby shutting down the expression of inflammatory cytokines including Tumor necrosis factor (TNF)-α, IFN-α, Interleukin (IL)-1β [19, 75].
(iii) The pathway to clear apoptotic cells also involves phosphatidylinositol 3-kinases (PI3K), phospholipase Cγ2, Src family kinases, and interactions with the αvβ5 integrin, etc.

TAM is a single transmembrane receptor (the orange part in the figure). From N-terminal to C-terminal (from top to bottom in the figure), there are three parts:1, the extracellular domain, including two IG domains (IG1, IG2) and two FNIII domains; 2, one single TM; 3, the intracellular domain, including one conserved PTK domain, one autophosphorylation site, and one ITIM domain [80, 91–94]. The intracellular PTK domain of TAM is the functional domain. TYRO3, AXL, and MER of TAM family all have phosphorylation and autophosphorylation sites, which can activate the corresponding signaling pathways by phosphorylation of downstream target proteins.

TAM does not directly bind to the PtdSer exposed on the cell surface but relies on GAS6 or PROS1 as a ligand [82]. GAS6/PROS1 consists of three parts (the blue part in the figure). From N-terminal to C-terminal (from top to bottom in the figure), there are N-terminal GLA domain, four EH -like domains, and C-terminal SHBG domain [34, 95, 96].

The TAM receptor binds to the C-terminal SHBG domain of GAS6/PROS1 through two N-terminal IG1 and IG2 in the extracellular domain. SHBG domain includes two tandem LG domains (LG1, LG2). The binding eventually leads to the activation of TAM receptor tyrosine kinase. GAS6/PROS1 binds to the PtdSer exposed outside the cell through the N-terminal GLA domain. With the assistance of vitamin K, the structure of the GLA domain can be stabilized through the binding of about 7 essential Ca^2+^ ions (yellow ball at the top in the figure) [34, 97–100]. The specific mechanism of interaction between the GLA domain and PtdSer will be introduced in section Vitamin K-dependent coagulation factors bind to PtdSer.

#### TAM, GAS6/PROS1 and PtdSer in blood coagulation system

When GAS6 ligands interact with TAM receptor family members (TYRO3, AXL, and MER) under hemostatic conditions, they can promote platelet aggregation to maintain thrombosis and platelet stability [25, 33, 81, 101, 102]. During this process, PtdSer will be exposed to activated platelets to participate in the production of thrombin [103] and will also bind to GAS6 to activate TAM [33]. PROS1 can be used as an anticoagulant in the anticoagulation process of Activated Protein C (APC) to inhibit coagulation. This anticoagulation process does not require the participation of TAM but must involve the participation of PtdSer (section The Cell-Based Model of Hemostasis involving PtdSer and Application of PtdSer in blood coagulation in this review). In addition, GAS6 was not found to be involved in the anticoagulation process of APC [104–107].

#### TAM and PtdSer in disease

Some diseases and pathological processes may involve the binding of TAM to PtdSer. For example, AD [7], Tumor and cancer such as breast cancer [22], DENV [15], EBOV [10–12], and RSV [16–18] are involved.

In AD, neuroinflammation stimulates microglia to produce superoxide, release nitric oxide, and then superoxide, which induces PtdSer exposure in neurons [7]. Furthermore, when exposed to soluble Aβ oligomers, microglia in adult brains engulf synaptic material through the CR3-dependent process [108]. These findings indicate that the PtdSer-exposed synaptic compartment can further result in excessive phagocytosis through the GAS6/PROS1/TAM receptor pathway and cause synapse loss in AD [109]. It may aggravate the symptoms of dementia such as aphasia, memory impairment, executive dysfunction, and behavior changes in AD patients.

As mentioned above, TAM receptors binding to PtdSer-exposed apoptotic cells by binding to the ligands GAS6/ PROS1 has a function of regulating immunity. In cancer, PtdSer is often exposed on the surface of tumor cells, tumor vascular endothelial cells, and tumor-derived vesicles [110–112]. At the same time, it is worth noting that the TAM receptor and their two ligands, GAS6/ PROS1, have been reported to abnormally express in various tumor microenvironments or promote tumor growth and infiltration [19, 47, 113–117]. It hints that the TAM receptor may play an immunological role in the tumor microenvironment through its ligand and PtdSer-exposed cells. Besides, Canan Kasikara et al., using TAM-IFNγR1 reporter lines and expressing TAM receptors in various epithelial cell models, revealed PtdSer regulates PD-L1 expression via TAM receptor, which fosters immune evasion and chemoresistance [118]. It indicates that PtdSer participates in the immune examination process in cancer through binging to the TAM receptor. In short, more researches are needed to illustrate the potential functions of the PtdSer, TAM receptors, and their ligands GAS6/ PROS1 in cancer.

Some viruses, such as Dengue [15], EBOV [10–12], RSV [16–18], can use multiple PtdSer receptors (e.g., TAM receptors) in our body to promote their attachment, entry, and replication in host cells through the interaction of bridge proteins such as GAS6/PROS1 with PtdSer [119].

### TIM receptors family recognizes PtdSer in the immune system

**Fig. 4:**
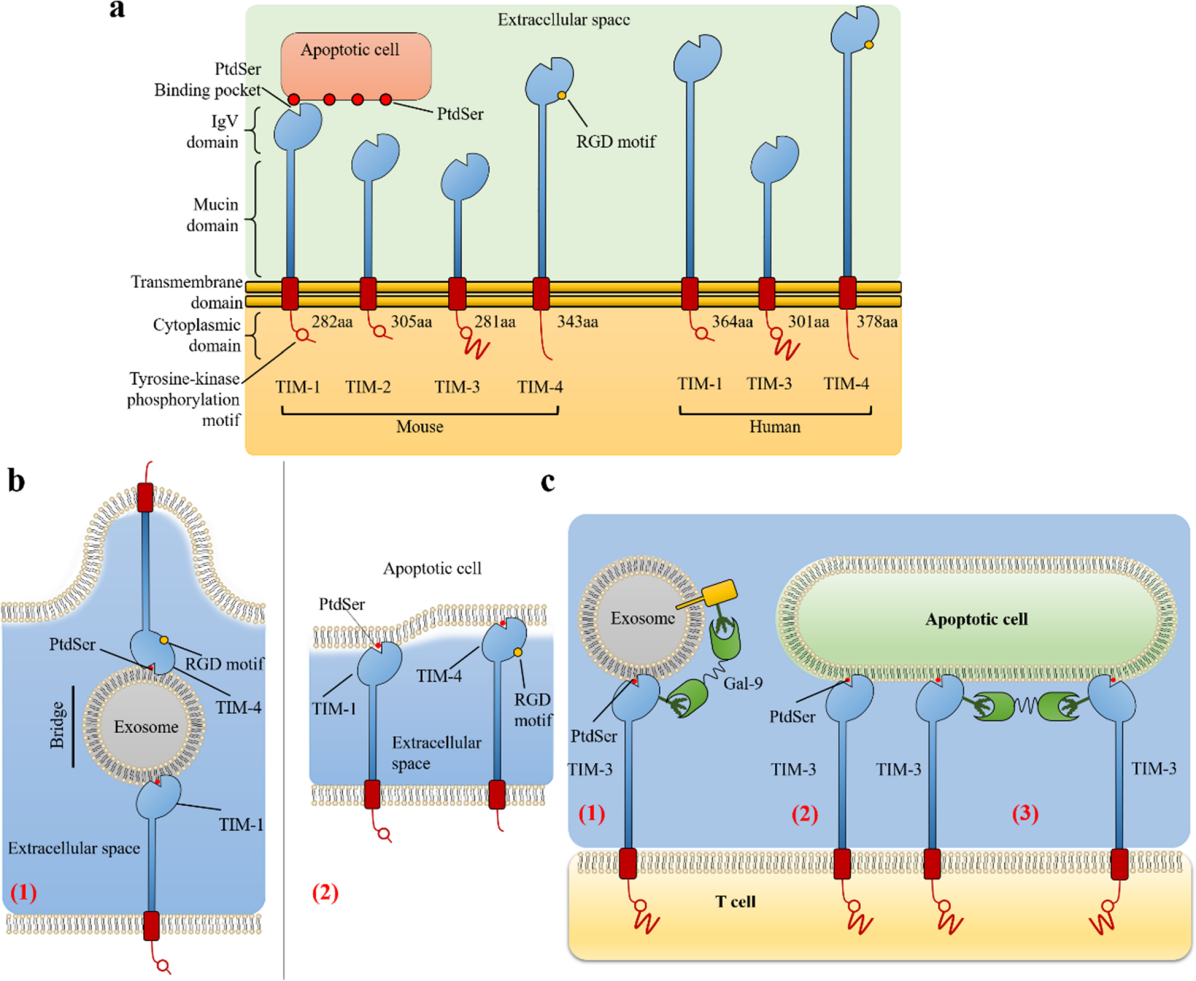
The structure of TIM and the binding to PtdSer. The structure of TIM family. In mice, there are TIM-1, TIM-2, TIM-3, TIM-4, but TIM-2 does not interact with PtdSer. In humans, there are only TIM-1, TIM-3, TIM-4 [8]. Only TIM-4 has an RGD motif in the IgV-like domain and lacks a tyrosine phosphorylation motif in the cytoplasmic tail. The RGD motif in TIM-4 is the ligand of integrins [35] and the hallmark for adhesive proteins [36–39]. The model of TIM-1 and TIM-4 interacting PtdSer. TIM-1 and TIM-4 molecules can interact with the PtdSer on the exosome to form a bridge [40]. (2) TIM-1 or TIM-4 can directly interact with PtdSer on apoptotic cells [39]. The model of TIM-3 interacting PtdSer. TIM-3 may interact with PtdSer on exosomes. Galectin-9 can also link TIM-3 to N-linked glycan on another protein [8]. (2) TIM-3 can directly interact with PtdSer on apoptotic cells [38]. (3) Two TIM-3 molecules can be cross-linked by Galectin-9 [41, 42]

The human TIM family includes three TIM proteins, including TIM-1, TIM-3, and TIM-4. They share a common structure, Immunoglobulin variable (IgV)-like domain that allows them to recognize PtdSer exposed on the surface of apoptotic cells with a high degree of specificity [35] (Fig. 4). TIM family regulates immune responses, including autoimmunity, transplant tolerance, the response to viral infections, and the regulation of allergy and asthma [120, 121].

Human TIM-1 (hTIM-1) is related to allergies, asthma, and autoimmune diseases, which indicates that TIM-1 regulates the immune system more comprehensively [35]. mTIM-1 (mouse TIM-1) is preferentially expressed in T helper 2 (Th2) cells, transmits signals that enhance T cell activation and proliferation, and increases airway hyper-reactivity and allergic reaction [122].

Tim-3 was originally thought to be expressed on mouse T helper 1 (Th1) and T helper 17 (Th17) cells. Also, cell subsets in the innate immune system express TIM-3, including human natural killer cells, monocytes [123] and dendritic cells [124], macrophages [125], and dendritic cells [126].

TIM-4 does not express in T cells. It expresses on antigen-presenting cells, such as monocytes-phagocytes, dendritic cells, B cells [127], iNKT cells [128], and even tumor cells [129].

#### TIM binds to PtdSer

Cells expressing TIM family members bind to or phagocytose apoptotic cells expressing PtdSer (Fig. 4 a) [127, 132, 133, 137].TIM-3 has various ligands, such as PtdSer and Galectin-9 (Fig. 4 c) [135]. Galectin-9 is an S-type lectin, which is a ligand of TIM-3. Galectin-9 binds to the carbohydrate chain on TIM-3, induces T helper 1 (Th1) cell death [135], and acts as a negative regulator of Th1 / T cytotoxic cell (Tc1) function [138]. The interaction between TIM-3 and PtdSer does not exclude the interaction with Galectin-9, because the binding site is located on the opposite side of the IgV domain [35]. The binding of PtdSer and TIM-3 phosphorylates tyrosine residues in the cytoplasmic tail of TIM-3. The binding is essential for TIM-3 signaling, but the downstream signaling mechanism in T cells is still unclear [139, 140]. It hints that the interaction of TIM-3 and PtdSer may induce apoptotic cell phagocytosis and antigen cross-presentation [137].

In phagocytes, TIM-4 can directly bind to PtdSer (Fig. 4 b (2)) [127] or indirectly through exosomes (Fig. 4 b (1)) [134]. Macrophages expressing TIM-4 recognize PtdSer of apoptotic cells, which leads to phagocytosis and clearance of apoptotic cells. However, the cytoplasmic domain of TIM-4 has only about 40 amino acids, which is too short to contain any tyrosine signaling motifs [35]. TIM-4 is considered to use the activity of TAM tyrosine kinase for signal transduction. TIM-4 alone cannot support phagocytose apoptotic cells but can significantly enhance the endocytosis mediated by the TAM signal system [141]. However, in the process of TIM-4 mediated endocytosis, the molecular mechanism of TIM-4 signal transduction to phagocytes is not fully understood to date. A recent study found that the physical interaction between TIM-4 and MER is necessary for MER to enhance TIM-4 mediated endocytosis. This physical interaction is mediated by the interaction between the IgV domain of TIM-4 and the fibronectin-like III domain (FNIII domain) of MER [142], which partially explains the mechanism above. Furthermore, TIM-4 is also considered to be the ligand of TIM-1. TIM-4 and TIM-1 can bind to each other with PtdSer on the Exosome to form a bridge. TIM-4 and TIM-1 may participate in cell-cell interactions through PtdSer on exosomes [134].

Additionally, exosomes expose PtdSer [143] on their outer leaflet and contain various cellular proteins, including galectins [144]. These extracellular vesicles are thought to be involved in many biological processes such as immune response and be involved in the communication between cells [145]. TIM protein may become one of the receptors of exosomes through PtdSer. Exosomes may bridge any two TIM proteins except TIM-2 (Fig. 4 b (1)) [134].

#### TIM and PtdSer in immune system

The binding of TIM family to PtdSer is a critical signal in the immune system. TIM-1 is constitutively expressed in Invariant natural killer T (iNKT) cells. Its recognition of PtdSer can induce iNKT cell activation, proliferation, and cytokine production [146]. At the same time, TIM-1 can also be highly upregulated on the surface of damaged renal epithelial cells, then giving epithelial cells the characteristics of semi-professional phagocytes. Apoptotic substances in the renal tubules can be removed by identifying and binding PtdSer on the surface of apoptotic cells [147]. Since TIM-1 can be expressed in injured and cancerous kidney samples, it is also known as kidney-injury-molecule-1 (KIM-1) in kidney and oncology [148]. So far, the role of PtdSer in regulating the function of TIM-3 is unclear. Studies have shown that the interaction between TIM-3 and PtdSer may participate in the phagocytosis of apoptotic cells and the subsequent cross-presentation of antigens [137]. TIM-4 on macrophages can bind to PtdSer on the surface of apoptotic cells to mediate the clearance of apoptotic cells [35]. However, the molecular mechanism by which TIM-4 transmits signals to phagocytes in the process of PtdSer and TIM-4 mediated endocytosis is not yet fully understood. TIM-4 is considered to require the tyrosine kinase activity of MER in the TAM receptor family for signal transduction [141].

#### TIM and PtdSer in disease

Many viruses invade cells by using the PtdSer exposed on their surface to interact with PtdSer receptors (such as TIM receptors), including HIV [149], EBOV [9], DENV [13–15], *etc*.

Acquired immunodeficiency syndrome (AIDS) is an immunodeficiency disease mainly caused by HIV infection [149]. Studies have found that multiple endocytic pathways promote the internalization of HIV-1 into epithelial cells. HIV-1 guides itself into the cell through interaction with PtdSer, TIM-1, heparan sulfate proteoglycans (HSPG), galactosylceramide (GalCer), and endocytosis and macropinocytosis [8]. TIM-1 also promotes HIV-1 entry into CD4+ T lymphocytes, However, the interaction between TIM-1 and PtdSer exposed on the surface of HIV during the release of progeny virions inhibits the release of the virus and retains the virus particles on the cell surface, thereby reducing the production and replication of HIV-1 [150].

EBOV, a member of the *Filoviridae* family of viruses, utilizes PtdSer receptors for entry into target cells. The PtdSer on the surface of the virus particle mediates the entry of filovirus through the conserved PtdSer binding pocket (MILIBS) in the amino-terminal IgV domain of TIM-1 and TIM-4. This discovery deepens understanding of the interaction between TIM-4 and EBOV virus particles [9].

Dengue is a viral disease transmitted by mosquitoes in tropical and subtropical regions. Studies have shown that TIM-1 can promote virus entry into cells during DENV infection and activate DENV autophagy [13]. DENV simulates apoptotic cells by exposing PtdSer on the viral membrane to promote the binding of TIM-1 [14].

In the tumor microenvironment, the interaction of PtdSer (exposed on cancer cells or tumor-derived exosomes) and PtdSer receptors (such as TIM-3 and TAM) expressed on immune cells can trigger a precisely-controlled-immunosuppressive pathway to weaken the innate and adaptive immune response and ultimately promote immune escape [19–21].

### Other immune receptors recognize PtdSer in the immune system

In addition to the TAM family and TIM family receptors, there are other receptors on the surface of macrophages, dendritic cells, and certain endothelial cells. For instance, αvβ3 or αvβ5 integrins [63, 64], CD300a [14, 65], BAI1 [66], Stabilin [67, 68], RAGE [69], LOX-1 [70], etc. They bind to PtdSer in a direct or indirect manner and recognize PtdSer on the surface of apoptotic cells, leading to endocytosis (Table 1).

**Table 1:** Immune receptors that recognize PtdSer

Immune Receptor	Cellular distribution	PtdSer recognition manner	PtdSer binding site	Function after binding to PtdSer	Disease (*represents related to PtdSer)
***TAM (TYRO3, AXL, MER)***	Macrophage, dendritic cells [74, 75], *etc*.	Indirect recognition	TAM ligand GAS6/PROS1 GLA domain [34, 97–100]	Identify apoptotic cells and prevent inflammation. Stable platelets [25, 80, 81]	*Virus invasion [10–12, 15], *Certain tumor and cancer [22], *Alzheimer’s disease [7, 109], SLE [151], Rheumatoid Arthritis [152]
***TIM (TIM-1, TIM-3, TIM-4)***	T helper 2 [122], T helper 1 [136], macrophage [127], *etc*.	Direct recognition	IgV-like domain [35]	Identify apoptotic cells and promote endocytosis [35]	*Virus invasion (HIV [8], Ebola virus [9], Dengue^12^, *etc*.) *Tumor [19–21]
***αvβ3 or αvβ5 integrins***	Macrophage [153]	Indirect recognition	αvβ3 or αvβ5 integrins ligand MFG-E8 C2 domain [154, 155]	Endocytosis, mediates related signal transduction [63, 64]	SLE [156]
***CD300a (human and mouse )[***14***,*** 65***], CD300b(mouse )*[**157**],** ***CD300f (mouse )*[**158**],** ***CD300c(human )*[**159**]**	Macrophage (CD300b) [157], myeloid and lymphoid cells [160]	Direct recognition (CD300a)	IgV-like domain (CD300a)	Promote phagocytosis of apoptotic cells (CD300b) [157]	*Dengue virus [14] Infectious diseases, cancer [161]
***BAI1***	Macrophage [66]	Direct recognition	TSP-1 [66]	Promote maximum phagocytosis of apoptotic cells [66]	*Bacterial infections [162] Medulloblastoma, glioblastoma [163]
***Stabilin (stabilin-1 and stabilin-2)***	Macrophage [164, 165]	Direct recognition	EGF-like domain [67, 165]	Endocytosis [164, 165] and cell fusion [166]	*Sepsis [167]
***RAGE***	Macrophage [69]	Direct recognition	IgV-like domain [168]	Induce phagocytosis [69]	diabetes [169], cancer [170], alzheimer’s disease [171], a potential inflammatory mediator of SARS-COV-2 infection [172]
***LOX-1***	Macrophage [70]	Not sure yet	Not sure yet	Endocytosis [70], Ingest oxidized low-density lipoprotein [173]	Atherosclerosis in patients with SLE [174], cancer and tumor [175]

## The mechanism of PtdSer exposure on activated platelets in the blood coagulation system

Under normal circumstances, platelets isolate the aminophospholipids, PtdSer and PtdEtn, in the inner leaflets of the membrane, while the PtdCho preferentially occupy the outer leaflets of the membrane [176]. This asymmetry is also maintained by flippase (such as ATP11C and ATP11A), which can transport PtdSer and PtdEtn from the outer leaflets of the plasma membrane to the inner leaflets [177]. When the tissue cells are not damaged, the coagulation factor mainly contacts choline phospholipids on the blood cells. These phospholipids constitute a non-thrombogenic surface [178]. Damaged tissue cells activate platelets, therewith the level of Ca^2+^ in the platelet cytoplasm increases which triggers changes in the lipid composition of the platelet membrane as PtdSer externalization [179]. Once exposed to the surface of platelets, PtdSer binds to various coagulation factors and promotes their enzymatic activity, eventually leading to the massive production of thrombin. The thrombin is indispensable for forming clots in damaged tissue cells, and the resulting clots can achieve hemostatic effects [28–32, 103]. Therefore, the normal exposure of PtdSer is extremely important for the subsequent generation of thrombin, thereby triggering the hemostatic effect.

### Increased Ca^2+^ concentration activates the activity of TMEM16F and inactivates ATP11C and ATP11A

**Fig. 5:**
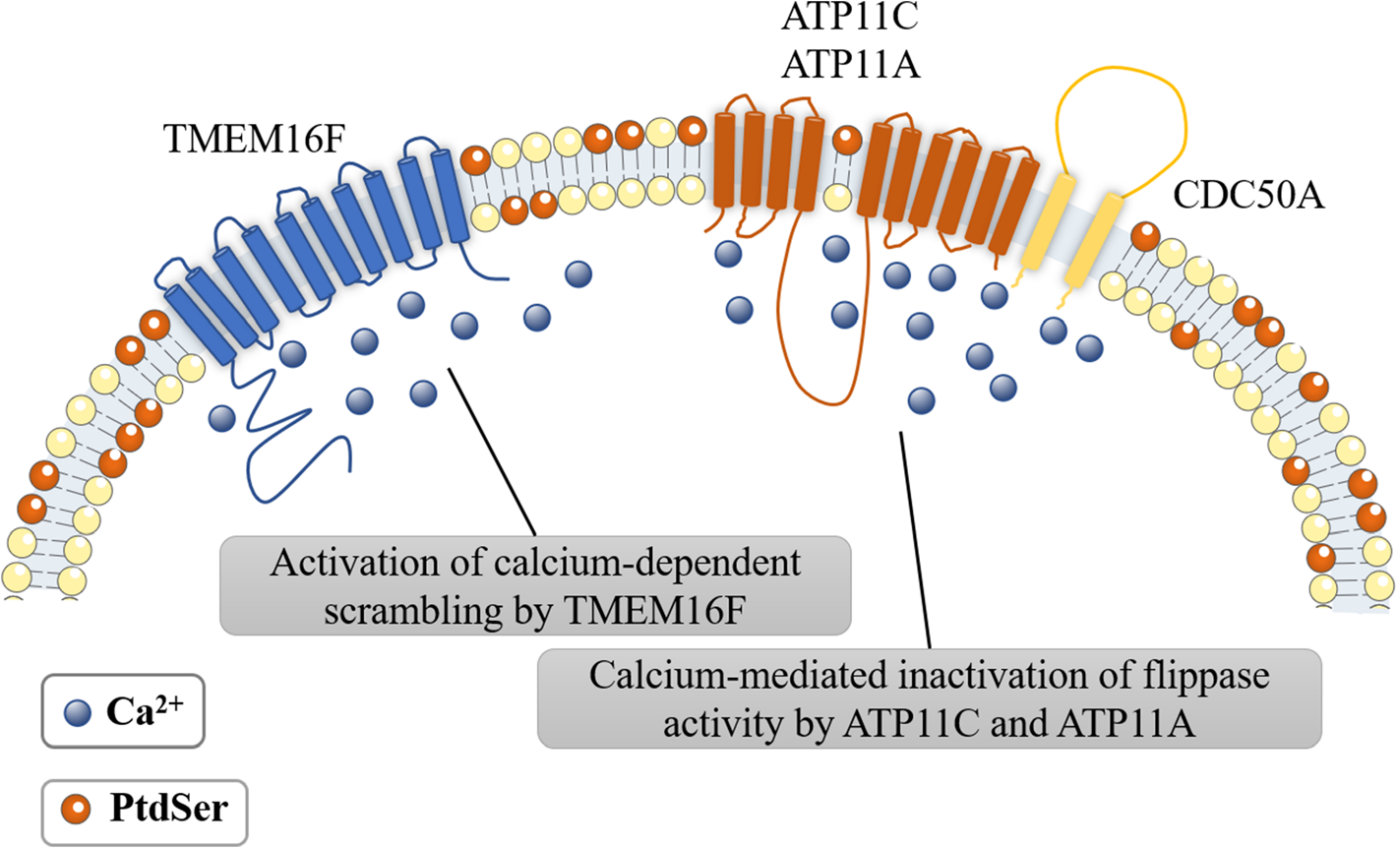
The model of PtdSer exposure on the surface of activated platelets. In the plasma membrane, flippases (ATP11C and ATP11A) specifically flip PtdSer from the outer leaflet of the lipid bilayer to the inner leaflet, forming an asymmetric distribution on the plasma membrane [43], while scramblase TMEM16F is inactive [44]. Once platelets are activated by thrombin, the concentration of Ca^2+^ in the activated platelets increases temporarily [45], instantly activates the scramblase activity of TMEM16F [44], inactivates the flippase activity of ATP11A and ATP11C [41], and quickly expose PtdSer on the surface of platelets. Once the platelets return to the normal state, the level of Ca^2+^ in the platelets decreases, which inactivates TMEM16F, restores the flippase activity of ATP11A and ATP11C [43], and re-establishes the asymmetric distribution of PtdSer in the plasma membrane. In short, Ca^2+^-induced PtdSer exposure is reversible

TMEM16F belongs to the TMEM16 family and is a Ca^2+^-dependent PtdSer scramblase as mentioned above [50]. TMEM16F, an ion channel, is essential to activate Ca^2+^-dependent externalization of PtdSer in platelets [19, 180, 181] (Fig. 5). It is comprised of ten transmembrane helices (TM). In the absence of Ca^2+^, its hydrophobic residue F518 of TM4 and hydrophobic residue I612 of TM6 will be close to each other to prevent the phospholipid head group from approaching the hydrophilic cavity; when Ca^2+^ binds to the hydrophilic cavity, the conformation of the cavity changes, which may cause TM6 to move around the conservative glycine hinge and rearrange TM3, TM4, and TM5, eventually leading to the separation of TM4 and TM6. Subsequently, the inside of the hydrophilic cavity is exposed to the surrounding phospholipids, and the phospholipid head group can enter the cavity, thereby activating TMEM16F [182]. In summary, the mechanism of Ca^2+^ activating TMEM16F is to change the conformation of the hydrophilic cavity of TMEM16F so that the hydrophilic cavity can be exposed to bind to the phospholipid head group. In the absence of Ca^2+^, the hydrophilic cavity will be closed by residues on the protein, preventing the hydrophilic cavity from binding to the phospholipid head group. Scott syndrome is a rare bleeding disorder causing increased epistaxis after trauma, often occurring after tooth extraction, and causing severe Postpartum hemorrhage [183]. Studies revealed it caused by insufficient Ca^2+^-dependent PtdSer exposure in activated platelets and mutations in the TMEM16F gene can cause it as well [3, 50].

Inactivation of ATP11C is also a Ca^2+^-mediated manner, which induced by activation of Ca^2+^-dependent protein kinase C (PKCα) [185]. PKC phosphorylates Ser1116, in the C-terminal cytoplasmic region of ATP11C, which leads to the production of the di-leucine sequence (pSVRPLL), corresponding to Leu1120 and Leu1121. The resulting pSVRPLL can be used for endocytosis [185]. Adaptor protein complex 2 (AP-2) is a key protein in clathrin-mediated endocytosis [186]. AP-2 can recognize two main endocytosis motifs, one of which is the acidic di-leucine motif [187, 188]. ATP11C is endocytosed by clathrin-mediated endocytosis, subsequently the flippase activity decreasing [185].

So far, there is no precise mechanism to explain the principle of Ca^2+^ inactivating ATP11A. However, studies have shown that Ca^2+^ can indeed inactivate ATP11A in the process of PtdSer exposure on platelets [41, 185]. Although ATP11A, like ATP11C, contains a sequence similar to the dileucine signal in its C-terminal cytoplasmic region, this sequence cannot act as an endocytosis signal through the Ca^2+^-mediated PKC activation pathway [185]. Therefore, the mechanism of Ca^2+^ inactivating ATP11A is not the same as that in ATP11C.

## PtdSer involved in the process of blood coagulation and anticoagulation

### The cell-based model of hemostasis involving PtdSer

**Fig. 6:**
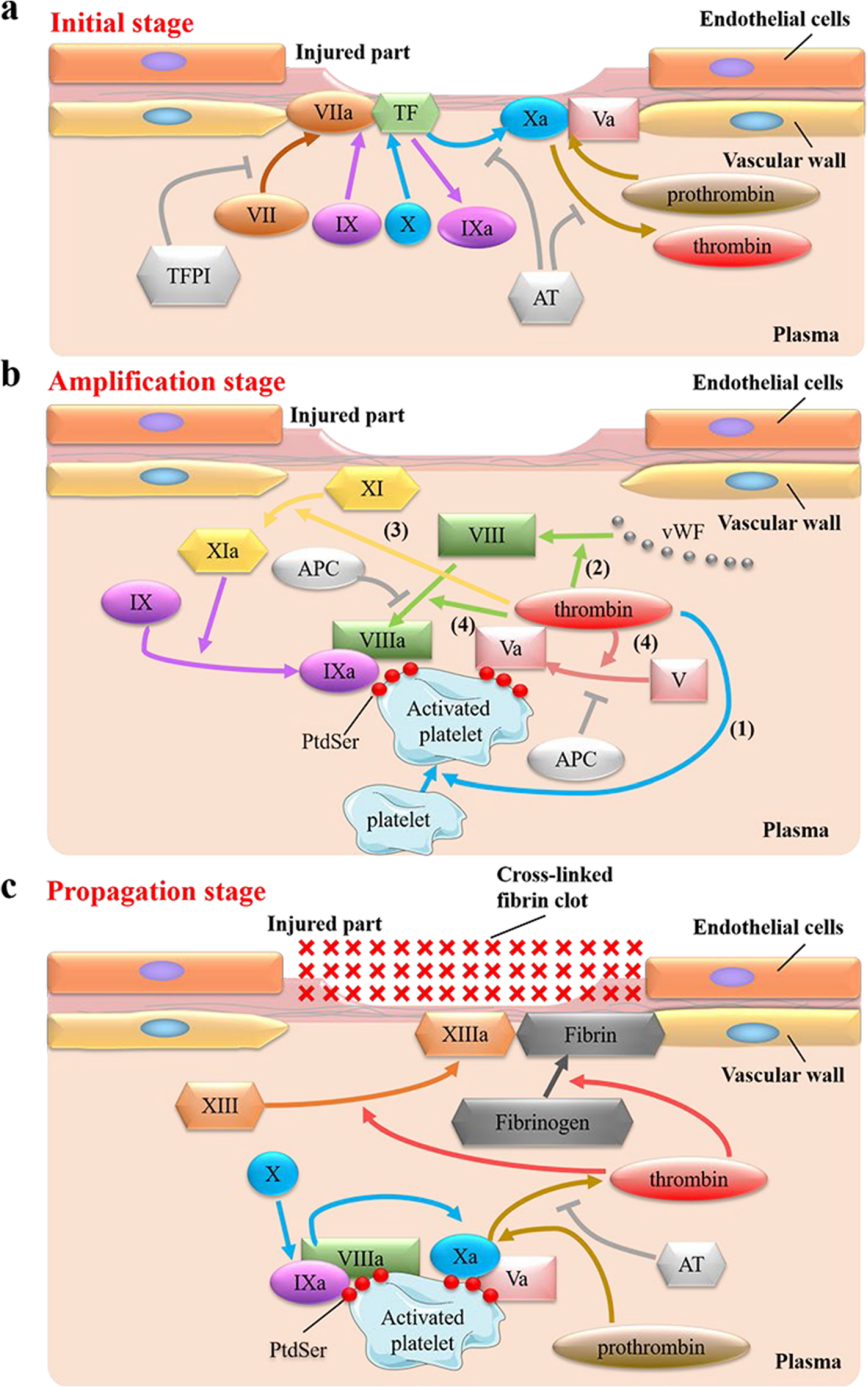
The Cell-Based Model of Hemostasis. **a** Initial stage of hemostasis. Plasma factor VII (FVII) binds to tissue factor (TF), then FVII automatically activates to FVIIa. On the surface of endothelial cells, FVIIa and TF together form a complex (FVIIa/TF complex). FVIIa/TF complex activates Factor IX (FIX) and Factor X (FX) to FIXa and FXa. On the same cell surface, FXa can form a Prothrombinase complex with its cofactor Va (FVa). The Prothrombinase complex can convert prothrombin (FII) to thrombin (FIIa). In this process, a small amount of thrombin is produced. However, a small amount of thrombin is not enough for hemostasis, and more thrombin needs to be generated further. **b** Amplification stage of hemostasis. The small amount of thrombin just generated has four functions in this process (the number here does not represent the order of reaction). (1) Thrombin activates the platelets in the plasma. The platelets are activated to form a thrombus formation surface. At the same time, PtdSer is exposed to the surface of the activated platelets. (2) Thrombin can also release Factor VIII (FVIII) from its carrier von Willebrand Factor (vWF). (3) Thrombin can activate Factor XI (FXI) to produce FXIa, then FXIa can activate Factor IX (FIX) to FIXa. (4) Thrombin activates Factor V (FV) to FVa and activates the newly released FVIII to FVIIIa. In these processes, the activation process of FIXa, FVa, and FVIIIa is carried out on the surface of newly activated platelets. At this time, on the surface of activated platelets, FIXa, FVa and FVIIIa are bound for the next stage of reaction. **c** Propagation stage of hemostasis. On the surface of activated platelets, FVIIIa and FIXa form a Tenase complex, which can activate FX to FXa. In addition, the rate at which Tenase complex activates Factor X to Factor Xa is faster than FVIIa/TF complex. FXa and FVa form a Prothrombinase complex, which allows prothrombin (FII) to be converted to thrombin (FIIa), resulting in massive production of thrombin - “thrombin burst”. PtdSer can promote the mass production of thrombin by binding with the Tenase complex and Prothrombinase complex. After “thrombin burst”, a large amount of thrombin can convert Fibrinogen (FI) to Fibrin (FIa) and can also activate Factor XIII (FXIII) to FXIIIa. FXIIIa can cross-link with FIa to form a Cross-linked fibrin clot, thereby forming a clot at the injured site to achieve hemostatic effect. Abbreviations: *TFPI* Tissue factor pathway inhibitor, *AT* Antithrombin, *vWF* von Willebrand Factor, *APC* Activated protein C

The latest cell-based hemostasis model [28–32] replaces the original coagulation cascade model [103] because the new model can better explain clinical hemostasis in vivo. The cell-based hemostasis model can be divided into three stages, initial stage, amplification stage, and propagation stage (Fig. 6). When the endothelial cells rupture, the tissue factor (TF) on the fibroblasts under the endothelium is exposed to the blood, which triggers the initiation of blood coagulation (Fig. 6 a).

During the coagulation process, after platelet activation, the content of PtdSer on the outer leaflets of platelets increases significantly. Tenase complex and Prothrombinase complex can interact with PtdSer to promote thrombin generation [103]. During the assembly process of Tenase complex (FVIIIa and FIXa) and Prothrombinase complex (FVa and FXa) (Fig. 6 b, c), FVIIIa and FVa act respectively as cofactors of FIXa and FXa [103]. Furthermore, Ca^2+^ and PtdSer are necessary for the function of these complexes. FIXa and FXa are vitamin K-dependent coagulation factors. They have a GLA domain, which binds to the phospholipids (such as PtdSer) on the platelet membrane in a Ca^2+^-dependent manner [189]. FVIIIa and FVa also require Ca^2+^ to stabilize their own connection after activation [190]. The light chains of FVIIIa and FVa bind to phospholipid membranes (such as PtdSer on the membrane), which is necessary to stabilize the protein after activation [191]. However, the binding of PtdSer and discoidin-like C2 domain in their light chain does not need to rely on Ca^2+^ [192]. There are three ways of anticoagulation to regulate blood coagulation.

(i) Tissue factor pathway inhibitor (TFPI) is a coagulation inhibitor that can regulate the initiation of coagulation induced by tissue factor (TF) (Fig. 6 a) [193].
(ii) Antithrombin (AT) is an enzyme that inhibits the coagulation system, which is a serine protease inhibitor (serpin) [194]. The physiological role of AT is to protect the circulation from the destruction of released enzymes and limit the coagulation process to the vascular injury site (Fig. 6 a, c). AT itself is an ineffective serine protease inhibitor. Heparin and heparinoid molecules on the surface of endothelial cells will activate it [195].
(iii) Another anticoagulant pathway is the PtdSer-dependent APC anticoagulant system. It inhibits the activities of FVIIIa and FVa (Fig. 6 b). In other words, it inhibits the function of the Tenase complex and Prothrombinase complex [196]. Thrombomodulin is a transmembrane protein of endothelial cells and peripheral blood cells, binds thrombin (FIIa) with high affinity, and changes its substrate specificity. The complex formed by thrombomodulin and IIa becomes an effective activator of zymogen protein C, which can activate zymogen protein C to APC [197]. APC can inactivate FVIIIa and FVa binding to PtdSer on activated platelets via a Ca^2+^-dependent manner, lead to the decomposition and inactivation of the Tenase complex and Prothrombinase complex, and inhibit blood coagulation.

### Vitamin K-dependent coagulation factors bind to PtdSer

Proteins FVII, FIX, FX, Prothrombin APC have a GLA domain at the N-terminal, which can bind to PtdSer in a Ca^2+^-depend manner and participate in the process of coagulation and anticoagulation [189]. In this process, vitamin K is necessary for the post-translational modification of GLA residues as well as the correct folding and activity [198]. So these coagulation factors can also be called vitamin K-dependent coagulation factors [103]. Also, in section TAM receptors family recognizes PtdSer in the immune and blood coagulation systems, it is mentioned that the ligand GAS6/PROS1 of TAM can also recognize and bind to PtdSer through the GLA domain, and GAS6/PROS1 is also vitamin K-dependent [34, 97–100].

The binding of the GLA domain to the PtdSer-exposed membrane is mediated by multiple interactions: Calcium coordination, ions, van der Waals forces, and hydrophobic interactions [100]. GLA domains from different coagulation factors show significant differences in lipid specificity and membrane binding affinity, which may depend on the interaction of non-conserved GLA domain residues of these coagulation factors with PtdSer [100, 103].

The GLA domain’s function is highly dependent on Ca^2+^ ions. Researches have established an atomic model of membrane binding GLA domains [199], showing that Ca^2+^ ions have two roles in the GLA domain: The four internal Ca^2+^ ions are mainly responsible for the correct folding of the GLA domain to insert into the membrane; the external Ca^2+^ ions anchor proteins on the membrane by directly contacting lipids. This model further indicates that Ca^2+^ is necessary for the GLA domain to bind to the membrane. Studies have shown that the GLA domain interacts with the negatively charged head group of the membrane phospholipid [199, 200]. More precisely, the phospholipid head group in the phospholipid (such as PtdSer) is bent so that the phosphate part of the phospholipid can form a coordination complex with the tightly bound Ca^2+^ in the GLA domain to performing their function**.** Furthermore, the stoichiometric ratio of the only complete PtdSer-GLA domain complex whose structure is determined by X-ray crystallography is 1:1, that in other words, one PtdSer molecule binding to one GLA domain [100].

Additionally, PROS1, one of the many coagulation factors consumed during the vigorous blood coagulation process, is a vitamin K-dependent anticoagulant protein as well [104, 105]. Its structure has been described in section TAM receptors family recognizes PtdSer in the immune and blood coagulation systems of this review. PROS1 is an essential inhibitory node in the coagulation reaction. In mice, congenital PROS1 deficiency can cause severe coagulopathy, leading to fatal coagulopathy and vascular dysplasia [106]. PROS1 can support the anticoagulant activity of APC. In human plasma, about 40% of PROS1 exists in the form of free protein S (FPS), while 60% of PROS1 forms a complex with C4b binding protein (C4BP). The complex causes the loss of the function of the PROS1 cofactor, and then C4BP can regulate the anticoagulant activity of APC [104]. PROS1 can also directly bind FXa and FVa to inhibit coagulation [25].

### The discoidin-like C2 domain of FV and FVIII binds to PtdSer

**Fig. 7:**
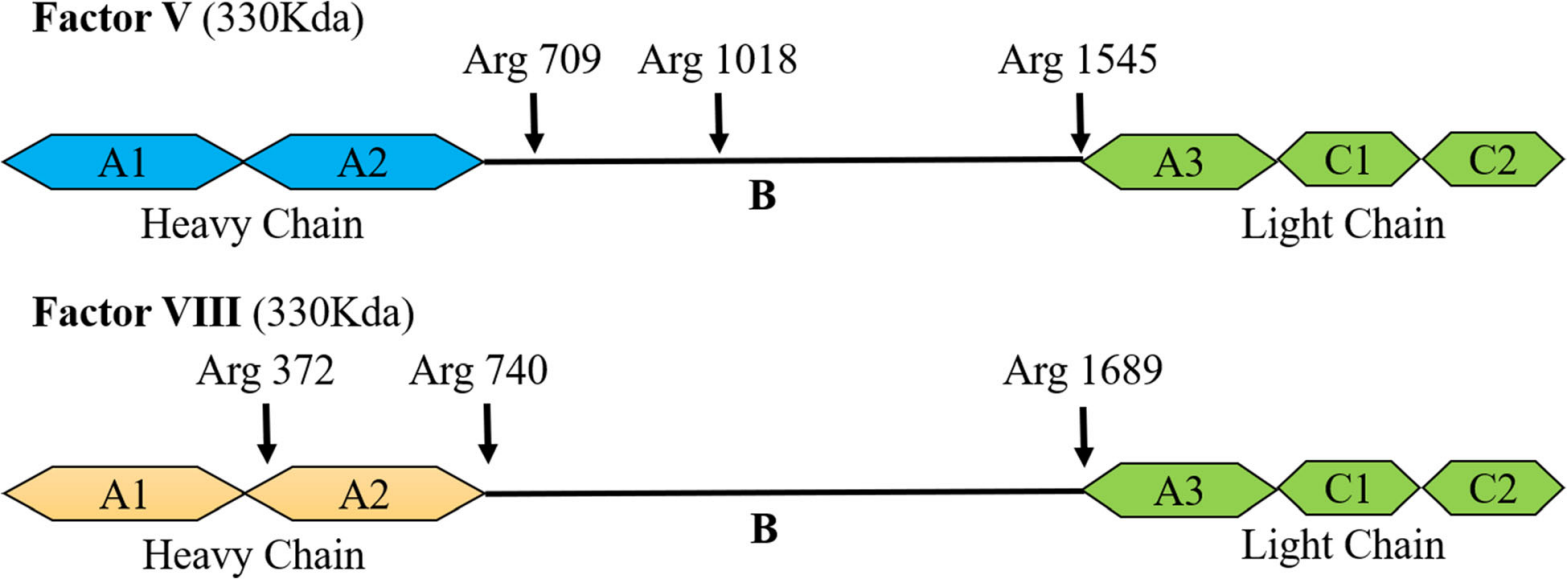
The structure of Factor V and Factor VIII. The figure indicates the cleavage site of Factor V and Factor VIII. After cleaved, Factor V and Factor VIII convert into their active form.Factor V and Factor VIII have the same sequence homology in their three A domains (A1, A2, A3) and two C domains (C1, C2), but entirely different sequence homology in their B domains. Both of them are converted by thrombin into their activated form (Factor Va and Factor VIIIa) [46]. Thrombin cuts off the non-homologous B domain, producing a heavy chain (A1-A2) and a light chain (A3-C1-C2), which are non-covalently combined into a heterodimer and stably connected by Ca2+ (For Factor VIII, A1 and A2 also need to be cut once more to form a heterotrimer) [47]. Their heavy chains contain the properties of cofactors that promote the activity of the Prothrombin complex and Tenase complex [48]. The light chains bind them to phospholipid membranes (such as PtdSer on the membrane). The light chains are necessary for stabilizing proteins after activation [49]

The PtdSer recognition site of FVa and VIIIa is located in their N-terminal C2 domain [201, 202]. The light chain and lipid interactions of FVa and FVIIIa involve electrostatic and hydrophobic binding [203, 204]. Their light chains have the same domain homology, consisting of an A-type domain and two smaller C-type domains (Fig. 7). Their C2 domain is a β-barrel core with three relatively long loops (also called “spikes”) protruding from one end [205, 206]. Deleting the C2 domain from recombinant FV completely eliminates PtdSer-dependent binding, while mutants containing only the C2 domain (lack of A3 and C1 domains) can still bind to PtdSer [201]. The C2 domain of FVa contains a soluble phosphatidylserine (C6PS) binding pocket [207]. There is a pair of tryptophan residues (Trp ^2063^ and Trp^2064^) in this lipid-binding pocket. Mutating these Trps eliminates the binding of FV to the membrane, indicating that these two Trp residues are necessary for the binding of FVa to PtdSer [208]. Some researchers have also shown that conservative mutations in the C2 domain of FVIII and FV can alter phospholipid binding and cofactor activity [209] and the Trp^2313^-His^2315^ fragment of the C2 domain of FVIII is involved in membrane binding [210]. All these indicate that the C2 domain of FVIII and FV is essential for PtdSer binding.

### Application of PtdSer in blood coagulation

In recent years, with the development of the coagulation mechanism, more and more diseases, such as essential hypertension (EH) [5], Hemophilia A [6], can be detected or treated by the interaction between PtdSer and coagulation factors.

The PtdSer exposed on the surface of platelets facilitates the assembly of Tenase complex and Prothrombinase complex, thereby promoting the production of thrombin on the surface of activated platelets [103]. Furthermore, Emily C. Reddy et al. shows that procoagulant PtdSer-exposing platelets can be used as an antithrombotic target. Inhibition of procoagulant platelet formation may be an alternative method to reduce thrombosis without compromising hemostasis [213].

Thrombosis is the main cause of death in patients with EH [214]. Studies have found that inflammatory cytokines can enhance the procoagulant activity of platelets and endothelial cells through PtdSer exposure in patients with essential EH. Therefore, PtdSer blockade may be a feasible treatment strategy for such patients [5].

Hemophilia A is a genetic disease. It is a serious bleeding disorder caused by mutations in the FVIII (also known as anti-hemophilia factor A (AHF)) gene on the X chromosome [215]. Studies have shown that the decrease in PtdSer expression on the platelet surface measured by Annexin V binding indicates an increase in preventive bleeding in patients with severe FVIII deficiency [6].

Recent studies have also found that PtdSer, exposed on the surface of some mesenchymal stem cells and their derived extracellular vesicles, potentiates blood clot formation. While the use of annexin V to block PtdSer on their surface will lead to their procoagulant activity partially lost [216]. Also, studies have shown that PtdSer, exposed to blood cells and microparticles in patients with non-valvular atrial fibrillation, enables them procoagulant activity, which leads to thrombosis [217]. Therefore, further researches are needed to explore the disadvantage of the procoagulant effects which caused by PtdSer exposure in the treatment of mesenchymal stem cells and in some diseases.

## PtdSer binding recognition site in the immune and blood coagulation system

Both GLA domain [82, 198, 218] and discoidin-like C2 domain [154, 205] can be used as PtdSer recognition binding sites during blood coagulation and immune processes. However, more PtdSer recognition and binding sites will be shared in the coagulation and immune process, which requires further experiment and research. It can be seen from the various recognition sites of PtdSer that some recognition sites (such as GLA domain) bind to the phosphate part of PtdSer [199, 200]. Therefore, not only PtdSer can interact with these domains, but PtdEtn and PA in phospholipids can also participate in the interaction with these domains in some processes (such as the recognition process with coagulation factors) [219, 220]. PA performs better than PtdSer in the APC-dependent anticoagulation pathway. It may be due to the X group of PA has smaller steric hindrance than that of PtdSer, leading to the better entry of these phospholipids into the GLA domain and binding to Ca^2+^. However, the relatively large choline group of PtdCho will hinder the binding of the PtdCho and GLA domain [219].

Discoidin-like C2 domain not only appears in coagulation factors (FV and FVIII) [201, 202] but also in the ligand MFG-E8 of immune receptor αvβ3 or αvβ5 integrin [154, 155]. Moreover, the discoidin-like C2 domains of these proteins have similar structures, and they are all involved in the binding with PtdSer [221]. Occasionally, protein kinase C (PKC) in the nervous system also has a PKC-C2 domain and participates in the binding of PtdSer in a Ca^2+^-dependent manner, thereby supports neuronal survival and differentiation in neuronal signal transduction [222, 223]. However, the PKC-C2 domain and discoidin-like C2 domain do not belong to the C2 domain though they are literally similar [224, 225].

There are IgV-like domains in some immune receptor proteins, like TIM family proteins [35], CD300 family proteins [14, 65, 226], and RAGE [168]. The IgV-like domains of TIM-1, TIM-3, TIM-4, CD300a, and RAGE are all involved in the binding of PtdSer and are Ca^2+^ dependent. It could be speculated that whether other proteins containing IgV-like domains also have the property of binding to PtdSer, which needs further study.

Besides these Ptdser recognition sites mentioned above (Table 2), there are many other binding sites of PtdSer. Annexin V can specifically recognize PtdSer, which needs the participation of Ca^2+^ [227]. Annexin V is widely used as a probe for detecting PtdSer on the cell surface [228–230]. In addition, TSP-1 repeats of BAI1 can bind to PtdSer in the extracellular area and promote the maximum phagocytosis of apoptotic cells through the ELMO-Dock180-Rac1 mechanism [66]. TSP-1 can also participate in many physiological and pathological processes, such as regulating PtdSer-dependent red blood cells in red blood cell-endothelial adhesion, which can be used as a potential therapeutic target [231]. Besides, the EGF-like domain is also a PtdSer recognition binding site. For example, Stabilin-1 and Stabilin-2 are binding with PtdSer through their EGF-like domain [67, 68] in endocytosis [164, 165], cell fusion, *etc* [166].

**Table 2:** PtdSer recognition sites related to immune or blood coagulation

PtdSer binding recognition site	Immune receptor or coagulation factor	Ca^**2+**^ dependence	Function after binding to PtdSer	References
**GLA domain**	GAS6/PROS1 ligand of immune receptor TAM	Yes	Identify apoptotic cells (GAS6/PROS1), stable thrombosis and maintain platelet stability (GAS6)	[33, 34]
	Coagulation factor vitamin K-dependent proteins (Factor VIIa, IXa, Xa, prothrombin and activated protein C)	Yes	Coagulation and anticoagulation	[107, 189]
**Discoidin-like C2 domain**	MFG-E8 ligand of immune receptor αvβ3 or αvβ5 integrins	No	Identify apoptotic cells	[63, 64, 154, 155]
	Coagulation factor Factor Va and Factor VIIIa	No	Maintain protein stability	[191]
**IgV-like domain**	Immune receptor TIM family (TIM-1, TIM-3, TIM-4)	Yes	Identify apoptotic cells and promote endocytosis.	[35]
	Immune receptor CD300a	Yes	Identify apoptotic cells	[35]
	Immune receptor RAGE	Not sure yet	Identify apoptotic cells	[69, 168]
**TSP-1**	Immune receptor BAI1	Not sure yet	Promote maximum phagocytosis of apoptotic cells	[66]
**EGF-like domain**	Immune receptor stabilin-1 and stabilin-2	Yes	Endocytosis and cell fusion	[67, 68, 164–166]

## PtdSer and PtdSer receptors may be involved in COVID-19

Corona Virus Disease 2019 (COVID-19) is a viral infectious disease caused by Severe Acute Respiratory Syndrome Coronavirus 2 (SARS-CoV-2), which poses a major challenge to the World Health Organization [232]. Acute inflammation and coagulation abnormalities appear to be the main cause of death for thousands of patients worldwide [233, 234].

Studies have speculated that PtdSer exposure may be involved in the SARS-CoV-2 infection of the outer leaflets of the cell membrane. PtdSer may be a potential mechanism or participant of inflammation and coagulation abnormalities in COVID-19 patients [23, 24]. As mentioned above, PtdSer can be exposed on the surface of platelets during the coagulation process to promote the explosive production of thrombin to promote coagulation [28–32, 103]. PtdSer can also be exposed on the surface of apoptotic cells during the immune process as an “eat-me” signal, recognized by receptors on the surface of phagocytes (such as TAM or TIM). Then the apoptotic cells are engulfed by phagocytes, thereby avoiding inflammation in our body [19, 35, 47, 49]. However, under pathophysiological conditions, some viruses, such as HIV [8], EBOV [9–12], DENV [13–15], and RSV [16–18], can use the PtdSer exposed on their surface to bind to PtdSer receptors on immune cells to invade cells, thereby increasing the infectivity of these viruses. In addition, study [235] has shown that bacterial endotoxins can cause PtdSer exposure to excessively activate coagulation, leading to life-threatening disseminated intravascular coagulation (DIC).

Similarly, some researchers [236] have shown that the GAS6/PROS1/TAM system is assumed to participate in SARS-CoV-2 infection and complications. In section TAM receptors family recognizes PtdSer in the immune and blood coagulation systems of this review, it is mentioned that TAM participates in hemostasis and anti-inflammatory [25, 80, 81]. GAS6/TAM can maintain thrombosis and platelet stability by promoting platelet aggregation [25, 33, 81, 101, 102], and PtdSer is also involved in this process. PtdSer will be exposed to activated platelets not only to participate in the production of thrombin [103] but also to bind to GAS6 to activate TAM [33]. Data reveals that in patients with severe COVID-19, the level of soluble AXL in GAS6 and TAM will increase [237]. Study speculates that TAM signaling plays a vital role in the coagulopathy associated with excessive inflammation observed in COVID-19 [236]. In addition, because some viruses, such as Dengue [15], EBOV [10–12], and RSV [16–18], can infect the host through using the PtdSer exposed on their own surface to interact with the GAS6/TAM of the host. Therefore, the study also predicts that GAS6, TAM, and PtdSer may participate in the invasion of the COVID-19 virus, which is an emerging and rapidly evolving situation [236].

Besides PtdSer, it was speculated that PROS1 might mechanically associate excessive blood coagulation with immune response in COVID-19 [238]. PROS1 can act as not only the activation ligand of TAM in the immune process [34, 83, 97–100], but also an anticoagulant in the blood coagulation process [104–106]. Furthermore, in these two processes, the two signal pathways cannot be activated without PtdSer [84, 107]. Study has shown that SARS-CoV-2 infection may be associated with explosive blood clotting [238]. The growing blood clots consume coagulation factors, including PROS1. Excessive consumption of PROS1 will not only cause abnormalities in the anticoagulation process but also unable the TAM receptors, especially MER, on immune cells to activate due to insufficient ligands. MER signaling will be silenced, resulting in inflammatory factors release of macrophages, such as IL-1β, TNF-α, etc. This study recommends testing the level of PROS1 during the standard blood test of COVID-19 patients. It shows that this measurement is beneficial for COVID-19 patients with coagulopathy and high D-dimer levels [238].

It has also been reported that the receptor for PtdSer, TIM-1 (KIM-1), may be the receptor for SARS-CoV-2 in the lung and kidney [239]. COVID-19 patients often have acute kidney injury [240], it has been demonstrated that TIM-1 is expressed in the epithelial cells of the lung and kidney of COVID-19 patients [239]. The enhanced expression of TIM-1 in human renal tubules increases the uptake of SARS-CoV-2 virions. It is also mentioned that many viruses, such as HIV [149], EBOV [9], DENV^12^, can use the PtdSer exposed on their surface to bind to TIM to enter host cells. Therefore, we suggested that therapeutic targeting for TIM-1 can prevent and/or treat COVID-19 and prevent the above virus from infected.

Based on various studies [236, 239], it can be assumed that PtdSer may be exposed to SARS-CoV-2 and then infected the host through the TAM and/or TIM system, but there is no sufficient evidence to prove this hypothesis. In addition, studies have shown that the symptoms of COVID-19 are mostly acute inflammation and excessive blood clotting [233, 234]. As mentioned above, TAM has a significant effect on anti-inflammatory in the human body, which needs the participation of PtdSer [34, 80, 241]. PtdSer also plays a major role in the blood system, such as the formation of thrombin [28–32, 103], the stabilization of platelets, the maintenance of thrombosis by the GAS6/TAM system [25, 33, 81, 101, 102], and the anticoagulation process that depends on APC and PROS1 [104–106]. These coagulation [103] and anticoagulation [107] processes all have the participation of PtdSer. In summary, based on the perspective of the mechanism of PtdSer on blood coagulation, immunity, and viral infection, further study of the relationship between PtdSer and COVID-19 may lay a solid foundation for discovering COVID-19 infection mechanism and detection/treatment methods.

## Conclusion and perspective

Immune systems and blood coagulation are vital for basic physiological functions. When tissues are injured, hemostasis is always the first step to control the injury. The hemostasis process usually requires platelets to complete. The cell-based coagulation mechanism has been explained in this article (section The Cell-Based Model of Hemostasis involving PtdSer). Blood coagulation and anticoagulation are very complicated processes involving the participation of various factors, and PtdSer is indispensable for activated platelets to produce thrombin [28–32, 103]. In addition, PtdSer plays a pivotal role in the Ca^2+^-dependent APC anticoagulant system [107, 196]. The lack of PtdSer on the surface of activated platelets will inevitably lead to a reduction in the production of thrombin, which will make the hemostasis process impossible to complete and cause a series of diseases. For example, Scott syndrome is a typical bleeding disorder caused by insufficient exposure of PtdSer in activated platelets [3].

Immune system is used to eliminate foreign antigens, or damaged cells, apoptotic cells, and tumor cells produced in our body to maintain homeostasis [242]. In this review, we mainly summarized the immune mechanism of macrophages to clear apoptotic cells responsible for innate immunity. Furthermore, PtdSer exposed to apoptotic cells is dominant for this innate immunity. For apoptotic cells to be successfully engulfed by macrophages, three conditions must be met:

(i) PtdSer is exposed on the surface of apoptotic cells. The exposure of PtdSer requires the activation of scramblase Xkr8 and the inactivation of flippase ATP11C and ATP11A. Caspase3/7 participates in the activation and the inactivation [40–44].
(ii) PtdSer receptors on macrophages can recognize and bind PtdSer normally. There are many PtdSer receptors on macrophages, including TAM receptor family (TYRO3, AXL and MER) [34], TIM receptor family (TIM-1, TIM-3 and TIM-4, 35], αvβ3 or αvβ5 integrins [63, 64], CD300a [14, 65], BAI1 [66], Stabilin [67, 68], RAGE [69], and LOX-1 [70], etc. Most of these receptors initiate endocytosis after recognized PtdSer on the surface of apoptotic cells. Moreover, TAM-mediated phagocytosis is the main mechanism to clear apoptotic cells [34].
(iii) PtdSer receptors can successfully activate downstream signal pathways after binding to PtdSer. For instance, the binding of TAM to PtdSer can activate Rac1 to activate phagocytosis [85] and inhibit the expression of inflammatory cytokine and NF-κB [19, 88]. It can also activate PI3K [25] and other signaling pathways. The recognition of TIM-1 and PtdSer can induce the activation, proliferation, and cytokine production of iNKT cells [146]. CD300b on macrophages can promote the phagocytosis of apoptotic cells through the DAP12-Syk-PI3K-AKT pathway after recognizing PtdSer [157]. After the PtdSer receptor BAI1 binds to PtdSer, it acts synergistically through ELMO / Dock180 / Rac to promote the maximum phagocytosis of apoptotic cells [66]. Further researches have explained and hypothesized the signaling pathways of many receptors binding to PtdSer. Although there are still many mechanisms maintain unknown, all the research results point to that PtdSer is a key link in activating related signal pathways.

The coagulation process which PtdSer participated in can be briefly summarized as follows: (i) When tissue cells are injured, a little thrombin will be produced. (ii) A small amount of thrombin can activate platelets, and the Ca^2+^ level in the activated platelets increases, which leads to the activation of scramblase TMEM16F and the inactivation of flippase ATP11C and ATP11A, thereby causing PtdSer exposure on the platelet cell membrane. Then, PtdSer binding to various coagulation factors (such as FVIIIa, FVa, FIXa, and FXa) on the surface of activated platelets will promote their enzymatic activity and causes a burst of thrombin production. (iii) A large amount of thrombin can activate Fibrinogen (FI) and FXIII (FXIII) to generate Fibrin (FIa) and FXIIIa. FXIIIa can cross-link with FIa to form a Cross-linked fibrin clot, which is very important for the formation of a blood clot at the injured site. The formation of blood clots can prevent excessive bleeding [28–32, 103, 179].

The successful elimination of apoptotic cells is closely dependent on each of the above parts. Particular diseases will occur due to the absence of individual steps. For example, PtdSer exposure failure may be the cause of SLE [4]. Apoptotic cells progress to secondary necrosis integrity when they are not cleared in an efficient and timely manner, which will lead the phagocytes to release pro-inflammatory cytokines (i.e., TNF-α, IL-1β), thereby inducing inflammation [49].

Besides eliminating apoptotic cells, the binding of PtdSer to immune cell receptors can lead to other immune functions. For example, inhibiting the expression of inflammatory factors and NF-κB inhibit inflammation [19, 88]. The recognition of PtdSer by TIM-1 can also induce iNKT cell activation, proliferation, and cytokine production [146].

However, some viruses (e.g. HIV [8], EBOV [9–12], DENV [13, 15]) can use the intersection between PtdSer and PtdSer receptors to enter the host. These viruses induce the virus and invade the host by using the binding of PtdSer on the viral membrane and the TIM or TAM receptor on the host’s immune cells. PtdSer on cancer cells can also bind to TIM-3 or TAM and promote immune escape [19–21].

Some factors and proteins in the process of coagulation and immunity have a similar structure (section PtdSer binding recognition site in the immune and blood coagulation system) that recognize and bind to PtdSer, such as GLA domain, discoidin-like C2 domain, IgV-like domain, etc. It is not excluded that other coagulation-related factors or immune receptors that have these PtdSer binding sites may also have the property of binding to PtdSer. More researches are needed and discovery to verify this hypothesis.

Since the coagulation and immune mechanisms have been continuously improved in recent years, many factors or receptors involved have been found. The relationship between the coagulation and immune mechanisms is being established [25, 26]. This review summarized the role of PtdSer in blood coagulation and immunity. Some studies have speculated that TAM [236, 243] and PROS1 [238] may participate in the most urgent COVID-19 in a non-canonical way. The symptoms of COVID-19 patients are mostly acute inflammation and excessive blood clotting [233, 234]. Due to the role of PtdSer in coagulation and inflammation, it has been speculated that it may be a potential PtdSer-related mechanism or participant in COVID-19 inflammation and abnormal coagulation [23, 24]. Studies [236, 239] have also assumed that PtdSer may be exposed to SARS-CoV-2 and then infected the host through the TAM and/or TIM system. Therefore, studying the intersection between PtdSer and COVID-19 may provide directions for discovering the mechanism of COVID-19 infection and detection/treatment methods.

PtdSer belongs to phospholipids on the membrane, and its importance for the biological membrane is obvious. PtdSer exposure is of great significance for blood coagulation [28–32, 103] and immunity [19], and PtdSer inside the cell is also indispensable to the nervous system [222]. Therefore, it is significant for the mechanism and treatment of concerning diseases to explore the mechanism and principles of PtdSer in blood coagulation and immunity.

## Data Availability

Not applicable.

## Abbreviations

AD: Alzheimer’s disease
AHF: Anti-hemophilia factor A
AP-2: Adaptor protein complex 2
APC: Activated protein C
AT: Antithrombin
BAI1: Brain angiogenesis inhibitor 1
BSG: Basigin
C4BP: C4b binding protein
Caspase: Cysteine-containing aspartate-specific proteases
COVID-19: Corona Virus Disease 2019
Cryo-EM: Cryo-electron microscopy
DENV: Dengue virus
DIC: Disseminated intravascular coagulation
EBOV: Ebola virus
EGF: Epidermal growth factor
EH: Essential hypertension
FI: Fibrinogen
FIa: Fibrin
FII: Prothrombin
FIIa: Thrombin
FIX: Factor IX
FN III: Fibronectin-like III
FPS: Free protein S
FV: Factor V
FVII: Factor VII
FVIII: Factor VIII
FX: Factor X
FXI: Factor XI
FXIII: Factor XIII
Gal-9: Galectin-9
GalCer: Galactosylceramide
GAS6: Growth-arrest-specific 6
GLA: γ-carboxyglutamate
HIV: Human Immunodeficiency Virus
HSPG: Heparan sulfate proteoglycans
IFN: Type I interferon
IgC-like: Immunoglobulin constant-like
Ig-like: Immunoglobulin-like
IgV-like: Immunoglobulin variable-like
IL: Interleukin
iNKT: Invariant natural killer T
ITIM: Immunoreceptor tyrosine-based inhibitory motif
KIM-1/TIM-1: Kidney-injury-molecule-1
LG: Laminin G
LOX-1: Lectin-like oxidized low-density lipoprotein receptor-1
MFG-E8: Milk fat globule epidermal growth factor 8
MILIBS: Metal ion-dependent ligand-binding site
NF-κB: Nuclear factor-κB
NPTN: Neuroplastin
PA: Phosphatidic acid
PtdCho: Phosphatidylcholine
PtdEtn: Phosphatidylethanolamine
PI3K: Phosphatidylinositol 3-kinases
PKC: Protein kinase C
PROS1: Protein S
PtdSer: Phosphatidylserine
pSVRPLL: di-leucine sequence
PTK: Protein tyrosine kinase
RAGE: Receptor for advanced glycation end products
RGD: Arg-Gly-Asp
Rho: Ras Homologue
RPE: Retinal pigment epithelium
RSV: Respiratory Syncytial Virus
RTKs: Receptor protein tyrosine kinase
SARS-CoV-2: Severe Acute Respiratory Syndrome Coronavirus 2
SHBG: Sex hormone binding globulin
SLE: Systemic lupus erythematosus
SM: Sphingomyelin
SOCS: Suppressor of cytokine signaling
TAM: Receptor Tyrosine kinases TYRO3, AXL and MER
Tc1: T cytotoxic cell
TF: Tissue factor
TFPI: Tissue factor pathway inhibitor
Th1: T helper 1
TIM: T cell/transmembrane, immunoglobulin and mucin
TLR: Toll-like receptor
TM: Transmembrane domain
TMEM16F: Transmembrane protein 16F
TNF: Tumor necrosis factor
TSP-1: Thrombospondin-1
vWF: von Willebrand Factor
Xkr8: Xk-Related Protein8
